# The roles of pleiotrophin in brain injuries: a narrative review of the literature

**DOI:** 10.1080/07853890.2025.2452353

**Published:** 2025-01-20

**Authors:** Yupeng Lei, Ruixi Zhou, Qian Mao, Xia Qiu, Dezhi Mu

**Affiliations:** a Department of Pediatrics, West China Second University Hospital, Sichuan University, Chengdu, China; b Key Laboratory of Birth Defects and Related Diseases of Women and Children, Sichuan University, Ministry of Education, Chengdu, China

**Keywords:** Pleiotrophin, brain injuries, neuroprotection, treatment, Z-type protein tyrosine phosphatase receptor

## Abstract

**Background:**

Pleiotrophin (PTN), a secreted multifunctional growth factor, is highly expressed in the developing brain. Recently, many studies have indicated that PTN participates in the development of brain and plays a neuroprotection after brain injury, especially promoting neuronal survival and neurite outgrowth, stimulating oligodendrocyte maturation and myelination, modulating neuroinflammation, and so on.

**Objective:**

However, no reviews comprehensively summarize the roles of PTN in brain injuries. Considering this, this review focuses on the roles and related regulatory pathways of PTN in brain injuries, what is known to date.

**Methods:**

PubMed and Embase databases have been searched, and related studies are compiled and summarized.

**Results:**

Our review has found PTN participates in the repairment of brain injuries, including hypoxic-ischemic brain injury, preterm white matter injury, traumatic brain injury, and neurodegenerative diseases, mainly based on animal data and small sample size studies. Besides, PTN interacts with receptors, such as, Z-type protein tyrosine phosphatase receptor and syndecan-3, regulating related pathways in these events.

**Conclusion:**

It suggests PTN as a promising candidate for the treatment of brain injuries clinically. However, the evidence is early in its development. Further multi-center and large-sample studies are warranted to support our findings and determine the clinical value of PTN for treating brain injuries.

## KEY MESSAGES

Our review has found PTN participates in the repairment of brain injuries, including hypoxic-ischemic brain injury, preterm white matter injury, traumatic brain injury, and neurodegenerative diseases. It suggests PTN as a promising candidate for the treatment of brain injuries clinically. Further multi-center and large-sample studies are warranted to support our findings

## 1. Introduction

Brain injury, one of the common disorders of central nervous system, can lead to death and disability, especially in the young individuals [1]. In the United States, approximately 1.7 million people suffer from brain injuries annually, which cause serious socio-economic burdens [1,2]. Brain injury, resulted from various causes and characterized by numerous pathogenic factors, has a complex pathogenesis [3]. The primary mechanism involves extensive cell death in the damaged brain tissues, which mainly driven by cerebral ischemia, hypoxia, inflammation, traumatic brain injury, etc [4,5].

Pleiotrophin (PTN) is a fibroblast mitogenic protein, which was first isolated and purified in 1989–1990 by researchers using different tissues, including bovine uterus, neonatal rat brains, and mouse bone tissue, etc [6–9]. The PTN gene is located on chromosome 6 among mice and chromosome 7 among human beings, separately. PTN is classified as a member of the secreted heparin-binding cytokine family by its high affinity for heparin. Studies have shown that, during the development of the brain tissues, PTN could support the survival, growth and maturation of neurons [10–12], and promote the differentiation, maturation and myelination of oligodendrocytes (OLs), as well [13]. Recently, as reported, PTN could reduce cerebral inflammation and promote the regeneration and repair of damaged nerves [14–16]. These findings suggest that PTN participates in the development of the brain tissues and plays a neuroprotection in brain injuries.

In clinic, PTN might be as a promising therapeutic target. Some reviews have reported PTN participates in neural repair and regeneration after peripheral nerve injuries [17,18]. However, there have no reviews been published about the roles of PTN in brain injuries. Hereby, this review describes the roles and related regulatory pathways of PTN in brain injuries, to find its potential value in the treatment of brain injuries.

## 2. The structure of PTN

PTN, a secreted multifunctional growth factor, binds strongly to heparin, also called heparin-binding growth-associated molecule [7,8]. PTN is an 18-KDa protein with 168 amino acids, of which, 136 amino acids make up the mature protein [18]. The amino acid sequence of PTN has a high evolutional conservation in human beings, rats, chickens, etc. Specifically, the homology is greater than 98% [19]. The human PTN gene is located on band q33 of chromosome 7 and consists of 5 coding sequences and 1 non-coding sequence [20]. The structure of PTN molecule contains the N- and C-terminal lysine-rich sequences and two beta-fold domains [21]. These two beta-fold domains jointly mediate interactions within and between cells, which could amplify biological effects [22]. Raulo E et al. indicated the synergistic action of beta-fold domains effectively promoted neurite growth and plasticity [23]. Furthermore, PTN is reported to participate in the development of brain and play a neuroprotection in brain injuries [14,15]. Paveliev M et al. found PTN enhanced dendrite regeneration in the cerebral cortex, by increasing with the density and number of the dendrites (*p* < 0.05) [14].

## 3. The expression of PTN in brain

During the development of brain, PTN is highly expressed in mitotically active cells. Studies have found that, during the embryonic and neonatal periods, PTN is highly expressed in the brain of rats, reaching its peak on the 7th day after birth; besides, PTN is highly and broadly expressed in the brain among newborn mice, and continues until the 12th day after birth, which corresponds to a critical period of brain development [8, 24,25]. Furthermore, on the 10th day after birth in mice, PTN is largely distributed in the subventricular zone (SVZ) of the lateral ventricles, and would reach an appearance peak, corresponding to the stage of oligodendrocyte differentiation and myelination [12, 26]. During the development of the brain, pericytes, neurons, and glial cells can express and secrete PTN [13, 27]. In addition, Qin EY et al. and Tang C et al. found that the adult neural stem and progenitor cells, located in the subgranular zone of the hippocampus and the SVZ of the brain white matter, could express PTN among adult mice [26, 28].

Furthermore, in the fetal human cerebellum, Santana-Bejarano MB et al. found Bergmann glia and granular cell precursors were mainly produced PTN at different gestational weeks, separately [29]. In the adult brain, only a few specific neuronal subpopulations in the cerebral cortex, cerebellum, hippocampus, olfactory bulb, and striatum can express and secrete PTN [25, 30,31].

## 4. The biological activities of PTN in brain

PTN, a neurotrophic factor, has multiple biological functions in brain, including promoting neuronal survival, supporting neurite outgrowth, inducing stem cell differentiation, stimulating oligodendrocyte maturation and myelination, and modulating neuroinflammation. Mean­while, some studies have reported PTN has several receptors, such as, Z-type protein tyrosine phosphatase receptor (PTPRZ), syndecan-3 (SDC3), glypican-2, anaplastic lymphoma kinase, N-syndecan, neuroglycan C, integrin avβ3, etc [32,33]. Simultaneously, PTN interacts with these receptors regulating related pathways (e.g. extracellular regulated protein kinases, phosphatidylinositol 3-kinase, and rho-associated coiled-coil kinase) in these biological activities among brain [32,33].

### 4.1. Promoting neuronal survival

PTN promotes the survival of neurons. Hida et al. showed that when embryonic midbrain neurons were cultured *in vitro*, the addition of PTN increased the number of tyrosine hydroxylase-positive neurons in a dose-dependent manner [34]. Furthermore, PTN increased the number of neurons and promoted their survival in cultured spiral neurons [35]. Nikolakopoulou et al. found that the addition of exogenous PTN could reduce neuron death caused by diphtheria toxin-induced ablation of pericytes [36].

### 4.2. Supporting neurite outgrowth

PTN supports neurite outgrowth. In zebrafish embryos, the overexpression of PTN induced neurite growth and complex branching [27]. An *in vitro* study found that, PTN secreted by neural stem cells promoted the morphological maturation of new neurons and neurite outgrowth [28]. During the perinatal period, neurons, astrocytes and oligodendrocytes express PTN, which was also showed to be an effective stimulator of neurite growth in the brain [37–39]. Furthermore, Tanaka M et al. and Li et al. demonstrated that PTN could engage with its receptor PTPRZ to regulate the dendritic morphology of postnatal neurons, and promote adult hippocampal neurogenesis [40,41].

### 4.3. Inducing stem cell differentiation

PTN induces stem cells to differentiate into neuron-like cells or neurons. An *in vitro* study by Yang S et al. demonstrated that PTN secreted by amniotic epithelial cells could induce human umbilical cord blood-derived mesenchymal stem cells to differentiate into dopaminergic neuron-like cells [42]. Additionally, PTN, in combination with stromal cell-derived factor 1, insulin-like growth factor 2, and hepatocyte growth factor B1, promoted the differentiation of human embryonic stem cells into functional tyrosine hydroxylase-positive neurons *in vitro* study [43].

### 4.4. Stimulating OLs differentiation, maturation and myelination

PTN promotes the differentiation, maturation, and myelination of OLs. Kuboyama K. et al. found that PTN treatment enhanced the differentiation of OL lineage cells into myelin basic protein-positive mature OLs within primary mixed glial cell cultures of mice [44]. Another *in vitro* study demonstrated that the addition of human recombinant PTN effectively stimulated myelination in primary OL cultures [45]. *In vivo*, Liu Z et al. found that PTN accelerated remyelination in lysolecithin-induced demyelinating mice [46]. Furthermore, they found that PTN promoted OLs differentiation and myelination, by specifically binding to PTPRZ1 receptor, then activating the downstream effector, p190RhoGAP [46]. By modulating Wnt pathway, PTN-PTPRZ signaling promotes proliferation among prenatal human OLs, reported by McClain CR et al. [47].

### 4.5. Modulating neuroinflammation

PTN is a potent modulator of neuroinflammation [48,49]. PTN potentiates lipopolysaccharide-stimulated microglia activation, which promotes neuroinflammation rather than suppresses [50]. Besides, PTN might interact with receptors PTPRZ to trigger tyrosine phosphorylation of Fyn kinase to participate in microglial neuroinflammatory processes [50]. Further experiments are needed to explore the PTN-related signaling pathways and molecules of downstream. However, Linnerbauer M et al. found PTN could reduce pro-inflammatory among both astrocytes and microglia [51]. This difference might be caused by acute or chronic brain inflammation.

## 5. The role of PTN in brain injuries

PTN actively exerts neuroprotective effects and participates in the repairment of brain injuries, including hypoxic-ischemic brain injury, preterm white matter injury, traumatic brain injury and neurodegenerative diseases [32, 34, 44–47, 52–65].

### 5.1. Hypoxic-ischemic brain injury

Hypoxic-ischemic brain injury (HIBI) arises from oxygen deprivation and/or ischemia of brain tissues. It frequently occurs in newborns, whose brains are underdeveloped and poorly tolerant to hypoxia and ischemia. Among children and adults, HIBI is commonly observed in instances of cardiac arrest.

In cases of HIBI with predominant cortical damage, PTN shows differential expression among different cell types in the cerebral cortex. Yeh HJ et al. indicated that in adult rat brain tissue subjected to focal ischemia, due to occlusion of the cerebral artery, the expression of PTN messenger ribonucleic acid (mRNA) significantly decreased in cortical neurons on the ischemic side, particularly more rapidly and markedly within the core ischemic area compared to surrounding regions [52]. Conversely, PTN mRNA expression increased in cortical astrocytes and microglia on the ischemic side. It suggests that PTN plays a vital pathophysiological role in recovering from ischemic brain injury.

Recently, one study focused on a rat model of HIBI induced by cardiac arrest and cardiopulmonary resuscitation showed that heparin treatment increased PTN expression, enhancing neurological functions and reducing brain edema, with water content in brain tissues reduced more than 10% (*p* < 0.01) [53]. Besides, in-vitro oxygen-glucose deprivation (OGD) model of primary neurons found that the heparin-mediated neuroprotection was significantly attenuated after inhibition of PTN and SDC3 expression. The PTN/SDC3 pathway was involved in heparin treatment for the cerebral ischemia-reperfusion injury [53]. Bertram S et al. found that the activation of PTN/SDC3 pathway could increase the number of spiral neurons (66.33 ± 6.96 vs. 19.67 ± 8.09, *p* = 0.04) and neurite length (1023.00 ± 74.07um vs. 221.30 ± 20.19um, *p* = 0.001), when compared with controls, as well [35]. In an in-vitro OGD model using microglial cells, Miao et al. demonstrated that exogenous PTN could activate the extracellular regulated protein kinases (ERK1/2) signaling pathway [54]. This activation promoted microglial proliferation and secretion of neurotrophic factors, like brain-derived neurotrophic factor (BDNF), nerve growth factor (NGF). Furthermore, PTN did not stimulate the expression of tumor necrosis factor-α and inducible nitric oxide synthase [54] (table 1). These findings suggest that PTN plays direct or indirect neuroprotective roles in HIBI. However, the evidence is early in its development. Further multi-center and large-sample studies are warranted to support the clinical value of PTN for HIBD.

**Table 1.** The roles of PTN in brain injuries.

The type of brain injuries	In-vivo model	In-vitro model	Treatment	Roles	Receptor	Mechanism	Reference
HIBI	cerebral ischemia-reperfusion injury following cardiac arrest and cardiopulmonary resuscitation	OGD of primary neurons	Heparin	enhance neurological functions, reduce brain edema, attenuate neuronal apoptosis and promote neurite outgrowth	SDC3	PTN/ SDC3 pathway	Liu W, et al. [53]
		OGD of microglial cell	PTN	promote microglial proliferation and secrete neurotrophic factors (BDNF and NGF)		ERK1/2 pathway	Miao J, et al. [54]
Preterm WMI	PTN-knockout mice	primary mixed glial cell	PTN	enhance the differentiation and myelination of OLs among PTN-knockout mice	PTPRZ		Kuboyama K, et al. [44]
	WMI neonatal rat model	OGD of OLN-93 cell line	recombinant PTN	improve the differentiation, maturation, and myelination disorder of OLs	PTPRZ	mTOR/YY1/Id4 pathway	Qiu X, et al. [19]
	Cys to Ser knock-in mice	OPC-like OL1 cells	PTN	enhance the differentiation of OLs	PTPRZ	PI3K/AKT pathway	Tanga N, et al. [64]
TBI	TBI mouse model	primary CNS neurons	PTN	enhance neurite outgrowth and promote dendritic regeneration in the cerebral cortex	glypican-2		Paveliev M, et al. [14]
	TBI mouse model	human iPSC-derived NPCs, iPSCs-derived neurons	recombinant PTN	promote neural stem cell proliferation and neurite growth			Qiu X, et al. [67]
PD		ventral mesencephalic culture	PTN	improve DA neuron survival and stimulate neurite length	PTPRZ or SDC3		Marchionini DM, et al. [76]
	6-OHDA-induced PD mouse model		PTN overexpression	increase the count of tyrosine hydroxylase-immunoreactive neurons			Taravini IR et.al [77]
	6-OHDA-induced PD mouse model		PTN overexpression	increase both neuron number in the substantia nigra pars compacta and neurite density in the striatum			Gombash SE et.al [78]
	hemi-PD rat model		PTN and dopaminergic neurons co-culture	promote the survival of dopaminergic neurons and the recovery of rotation function			Hida H et.al [79]
AD	senescence-accelerated mouse prone 8 mice	adult neural stem progenitor cell culture	PTN	improve learning- and memory-related cognitive function and ameliorate neurogenesis decline of mice	PTPRZ1	AKT pathway	Li H, et al. [41]

*Abbreviations:* PTN: pleiotrophin; HIBI: hypoxic-ischemic brain injury; OGD: oxygen-glucose deprivation; SDC3: syndecan-3; ERK1/2: extracellular regulated protein kinases; BDNF: brain-derived neurotrophic factor; NGF: nerve growth factor; WMI: white matter injury; OLs: oligodendrocytes; mTOR/YY1/Id4: mammalian target of rapamycin/YingYang1/inhibitor of DNA binding 4; PI3K/AKT: phosphatidylinositol 3-kinase/protein kinase B; iPSC: human-derived induced pluripotent stem cells; NPCs: neural progenitor cells; CNS: central nervous system; PD: parkinson’s disease; AD: Alzheimer’s disease; 6-OHDA: 6-hydroxydopamine.

### 5.2. Preterm white matter injury

White matter injury (WMI) is one of the most common types of brain injuries in preterm infants, typically presenting as diffuse WMI and impacting areas such as the white matter, cortex, and hippocampus, etc [55]. Brain white matter development relies on the differentiation, maturation, and myelination of OLs [56,57]. That is, abnormal development of OLs represent a primary pathophysiological alteration in WMI.

Recently, some studies have indicated that PTN actively contributes to the differentiation, maturation, and myelination of OLs [13, 27, 44–47]. Goebbels S et al. showed that the addition of human recombinant PTN effectively stimulated myelination in primary OL cultures [45]. An *in vivo* study showed myelination of OLs was delayed in the white matter regions of PTN-knockout mice [44]. Furthermore, exogenous PTN treatment decreased the differentiation deficits of Mea6-deficient OLs, by Ma T et al. demonstrated [58]. Notably, using a neonatal rat model which could mimic the pathologic alteration of WMI among human premature infants, our team found that PTN administration could improve the differentiation, maturation, and myelination disorder of OLs in the white matter areas among neonatal rats after WMI [19] (table 1). Besides, Morris water maze found that, when compared with rats in WMI group, the average latent escape time of rats was shortened (day5, 32.576 ± 22.628s vs. 17.292 ± 7.746s, *p* < 0.05) and number of platform crossings among rats was greatly increased (1.833 ± 1.850 vs. 3.917 ± 1.831, *p* < 0.05) in the WMI+PTN group, suggesting that PTN had a neuroprotective effect in rats with WMI [19].

Oligodendrocyte progenitor cells (OPCs), the primary source of myelinating OLs, abundantly express the PTN receptor (PTPRZ) [59,60]. PTN could enhance differentiation and maturation of OPCs by interacting with PTPRZ and deactivating it [44, 47, 61,62]. Extensive studies reveal that PTN binding to PTPRZ modulates potentially several downstream signaling pathways, like phosphatidylinositol 3-kinase/protein kinase B (PI3K/AKT), Rho/Rho-associated coiled-coil kinase, etc., thereby orchestrating the differentiation, maturation, and myelination of OLs [62–64]. Furthermore, our team supported mammalian target of rapamycin/YingYang1/inhibitor of DNA binding 4 (mTOR/YY1/Id4) signaling pathway was involved in the neuroprotective effect of PTN in neonatal rats with WMI [18]. The mechanisms of PTN regulation for OLs development are complex. However, the evidence is partially limited, based on largely animal model and small case study data above. Continued research could explore the roles and mechanisms of PTN for WMI, providing scientific evidence for treating preterm WMI clinically.

### 5.3. Traumatic brain injury

Traumatic brain injury (TBI) often occurs suddenly and is unpredictable, typically resulting from motor vehicle collisions or physical assaults. Paveliev et al. used a syringe needle to stereotactically puncture the cerebral cortex of adult mice, thus establishing an acute TBI model [65]. They further utilized this model to administer exogenous recombinant PTN directly into the site of cortical injury. Eventually, PTN persisted for over 20 days in brain and promoted dendrite regeneration in the cortical cortex [14]. A patent reported that exogenous PTN injection into the brains of adult mice with TBI promoted dendrite regeneration in neurons, as well [66]. In 2023, Qiu X et al. used the single-cell RNA-sequencing analysis to lucubrate TBI and revealed PTN was significantly enhanced in the subacute phase of TBI among mice (*p* < 0.05) [67, 68]. Besides, the expression of PTN in astrocytes was enhanced, promoting neural stem cell proliferation and neurite growth [67] (Table 1). Therefore, PTN may play a crucial role in spontaneous neuroregeneration. Further multi-center studies are warranted to support our findings.

### 5.4. Neurodegenerative diseases

#### 5.4.1. Parkinson’s disease

Parkinson’s disease (PD) is a common neurodegenerative disorder, characterized by progressive motor dysfunction and related to the deposition of α-synuclein [69,70]. With the progress of global aging population, the prevalence of PD has increased dramatically. In 1990, there were about 2.5 million PD patients, and the prevalence of PD increased by approximately 74% to 2016 [71,72]. The hallmark pathological change of PD is the extensive loss of dopaminergic neurons in the substantia nigra [73].

It is reported that PTN is up-regulated in the substantia nigra of PD patients, enhancing the survival of dopaminergic (DA) neurons, promoting neurite outgrowth, and providing significant nutritional support to DA neurons [17, 34, 74, 75]. Marchionini DM et al. found that PTN adding to ventral mesencephalic cultures could improve DA neuron survival and stimulate neurite length [76]. In 2011, Taravini et al. found that, in the substantia nigra of a 6-hydroxydopamine (6-OHDA)-induced PD mouse model, PTN overexpression increased the count of tyrosine hydroxylase-immunoreactive neurons [77]. Subsequently, Gombash et al. discovered that PTN overexpression could increase both neuron number more than 20% in the substantia nigra pars compacta and neurite density over 10 times in the striatum of a 6-OHDA induced PD mouse model [78]. These studies underscored the neuroprotective effect of PTN in PD. Additionally, research by Hida and colleagues demonstrated that co-culturing PTN with dopaminergic neurons and subsequent direct injection into the striatum of a PD model rat promoted the survival of dopaminergic neurons and the recovery of rotation function among rats [79] (Table 1). Levodopa, the first-line drug for PD, was found to increase the expression of PTN in the substantia nigra-striatal pathway of PD model rats [80]. All above, these studies support the potential value of PTN as a novel therapeutic approach for PD. In the dopaminergic system of the substantia nigra-striatum, PTN interacts with receptors PTPRZ and SDC3 to activate downstream signaling molecules (e.g. cadherins, cortactin, actin, and tubulin), and participates in regulating PD-related pathways [16, 32, 37, 76]. Hida H et al. showed that PTN/SDC3 signaling has a wide range of neurotrophic effects on DA neurons [34]. This interaction positions PTN as a promising candidate for advancing PD treatment in both preclinical and clinical settings.

#### 5.4.2. Alzheimer’s disease

Alzheimer’s disease (AD) is an exceedingly complex and widespread brain degenerative disorder, for which effective treatments are currently lacking [81]. Genetic and pathological evidence has shown that the accumulation of beta-amyloid in neurons, leading to senile plaques, is a primary characteristic of AD progression [82,83]. A proteomic analysis of cerebrospinal fluid from AD patients has revealed high expression levels of endogenous PTN peptides, proposing it as a novel biomarker for AD diagnosis [84]. In 2023, using a senescence-accelerated mouse prone 8 mice as a model of AD, Li H et al. found that replenishment of PTN could improve learning- and memory-related cognitive function and ameliorate neurogenesis decline of mice [41]. Mechanistically, PTN engaged with PTPRZ1, and activated AKT signaling, to enhance adult hippocampal neurogenesis and cognitive function [41] (table 1). It reveals a potential therapy by targeting PTN. Further large-sample clinical studies are warranted to determine the value of PTN for treating brain injuries.

## 6. Conclusion

In summary, PTN promotes neuronal survival, supports neurite outgrowth, induces stem cell differentiation, stimulates oligodendrocyte maturation and myelination, and modulates neuroinflammation. Meanwhile, PTN actively exerts neuroprotective effects and participates in the repairment of brain injuries, including hypoxic-ischemic brain injury, preterm white matter injury, traumatic brain injury, and neurodegenerative diseases. Additionally, PTN interacts with receptors, such as, Z-type protein tyrosine phosphatase receptor and syndecan-3, regulating related pathways in these events. It suggests PTN as a promising candidate for advancing the treatment of brain injuries clinically. Furthermore, it is necessary to make profound research on the potentially clinical value of PTN for treating brain injuries. However, above-mentioned are mainly based on animal data and small sample size studies, and the evidence is early in its development. Further multi-center and large-sample studies are warranted to support our findings and determine the clinical value of PTN for treating brain injuries.

## Data Availability

Data sharing is not applicable to this article as no new data were created or analyzed in this study.
